# Integrating TAM and uses and gratifications theory: how Chinese media content preferences predict YouTube-based Chinese language learning among Thai secondary school students

**DOI:** 10.3389/fpsyg.2026.1824902

**Published:** 2026-05-19

**Authors:** Binle Lai

**Affiliations:** Department of Education, Practice and Society, UCL Institute of Education, University College London, London, United Kingdom

**Keywords:** Chinese as a foreign language, informal language learning, technology acceptance model, Thai adolescents, uses and gratifications theory, YouTube

## Abstract

**Introduction:**

As Chinese entertainment media become increasingly popular among Thai adolescents, more and more students engage with such content on YouTube outside of school. However, the relationships among content preferences, perceived usefulness, behavioral intention, and self-perceived learning effectiveness have been insufficiently examined.

**Methods:**

This study combines the Technology Acceptance Model (TAM) and Uses and Gratifications Theory (UGT) to examine these associations in 266 secondary school students (aged 13–19) from two schools in Thailand. Structural equation modeling with bootstrap-based mediation testing was used to analyze data.

**Results:**

The results indicate that Chinese Media Content Preference (CMCP) positively predicted Perceived Usefulness (PU) (*β* = 0.593, *p* < 0.001), PU positively predicted Behavioral Intention to use YouTube to learn Chinese (BI) (*β* = 0.642, *p* < 0.001), CMCP directly positively predicted BI (*β* = 0.353, *p* < 0.001), and BI positively predicted self-perceived Learning Effectiveness (LE) (*β* = 0.766, *p* < 0.001). PU partially mediated the relationship between CMCP and BI, with an indirect effect comprising 74.22% of the total effect.

**Discussion:**

Overall, the results suggest that content-level gratification can be a significant external antecedent within the TAM framework, with perceived usefulness as the key mechanism linking content preference to use intention. The research provides implications for Chinese-language teachers and content developers who want to utilize entertainment media to teach language.

## 1 Introduction

In recent years, international Chinese-language education has shown a remarkable global growth and greater institutionalization. Based on data published by the Ministry of Education of China in September 2024, 85 countries have included Chinese in their national education systems, and the number of active international Chinese-language learners and users has exceeded 200 million (Ministry of Education of the People’s Republic of China, 2024). Meanwhile, the Confucius Institute and Confucius Classroom network have continued to expand, with course offerings and instructional services growing steadily (Chinese International Education Foundation, 2025). In 2024, the number of candidates sitting the global Chinese Proficiency Test (HSK) exceeded 810,000, representing a year-on-year increase of 15% (Center for Language Education and Cooperation [CLEC], 2025), representing the rapidly rising demand for formal certification of Chinese-language competence. All these indicators show that international Chinese learning is moving from sporadic, interest-based study to large-scale educational practice. In this global context, Southeast Asia stands out as one of the most active regions, and the Confucius Institute network has now covered the region’s major countries (Chinese International Education Foundation, 2025).

Chinese-language education in Thailand is the most institutionalized among Southeast Asian countries, as reflected in the consistency of supply-side support systems and the clear inclusion of Chinese in the basic education curriculum and the national talent development strategy. The Bureau of International Cooperation (BIC) of the Ministry of Education of Thailand reports that China has created 16 Confucius Institutes and 11 Confucius Classrooms in Thai higher-education institutions. In addition, the Center for Language Education and Cooperation sends approximately 1,000 to 1,500 Chinese-language volunteer teachers to Thai schools at all levels annually (Bureau of International Cooperation, Ministry of Education of Thailand, 2023). At the 2025 World Chinese Language Conference, Suthep Kaengsanthia, Permanent Secretary of the Ministry of Education of Thailand, also stated that Thailand has officially incorporated Chinese into its basic education curriculum, making it a language of opportunity and stating a strategic objective of developing trilingual competence in Thai, English, and Chinese (Bureau of International Cooperation, Ministry of Education of Thailand, 2025). This implies that the Chinese-language education in Thailand no longer exists as an independent foreign-language course but rather as an institutional structure entrenched in the logic of governance of basic education and the goals of talent development in the country.

Nevertheless, a high level of institutionalized classroom provision does not necessarily mean that learning only takes place in formal educational institutions. Formal curricula often provide learning goals, fundamental skills, and long-term motivation, but learners often structure their own learning spaces and materials outside the classroom to expand input and practice (Benson, 2011). The digital environment in Thailand offers positive conditions for extracurricular extension: the Internet penetration among 15- to 24-year-olds stands at 99.1% (National Statistical Office of Thailand, 2023), and a nationally representative survey of 113 schools and 23,659 secondary-school students found that 94.0% reported current YouTube use (i.e., within the last 30 days), making it the most widely used video platform among this age group (Jayuphan et al., 2026).

One of the numerous digital platforms is YouTube, which has become a major platform in language-learning research due to its structural affordances that make it unique among sites focused on real-time social interaction. The site provides a searchable content library, enables learners to interact with language input at their own pace, and offers multimodal scaffolding via captioned video. Willoughby and Sell (2024) conducted a longitudinal qualitative study that identified systematic use of online video as a second-language learning tool outside the classroom by the learners who demonstrated recognizable strategic patterns and learning paths; they observed that online video sources could provide authentic language input and help learners choose content and level of difficulty based on their needs, thus enabling them to learn languages independently. Even though most of this evidence comes from general second-language learning situations, the mechanisms behind multimodal video input, learner controllability, and caption scaffolding can be applied equally well to out-of-class learning in which Chinese is the target language. On an empirical basis, Arndt and Woore (2018) conducted an experimental study using YouTube vlogs as the materials. They discovered that watching such videos facilitated incidental acquisition of second-language vocabulary, and that video input was more successful on selected measures of semantic recognition and grammatical-function recall. Moreover, a meta-analysis of 18 studies carried out by Montero Perez et al. (2013) showed that captioned video input has significant and meaningful positive impacts on second-language listening comprehension and vocabulary acquisition, indicating that the multimodal characteristics of video platforms, the simultaneous display of visual imagery, audio speech, and textual captions, may offer useful scaffolding during the process of comprehension and vocabulary acquisition. In line with this, the main question of this study is not whether Thai students watch YouTube to learn Chinese, but rather how they assess and choose Chinese-language learning content on YouTube, how this assessment translates into further use intention, and how this intention finally relates to their self-perceived Learning Effectiveness (LE).

This study adopts a combination of TAM and UGT as its theoretical framework, developing an explanatory model of Thai adolescents’ behavior in learning Chinese through YouTube. The two theories provide complementary views on the relationships between content choice by learners, perception of technology, behavioral intention, and effectiveness of learning.

Technology Acceptance Model (TAM). One of the most effective theoretical models that explains individual technology adoption is TAM, developed by Davis (1989). Perceived Usefulness (PU) is a direct predictor of Behavioral Intention (BI) in many empirical applications, representing the essence of the acceptance mechanism, where users are more willing to use a technology when they perceive it as contributing to task performance. The present study does not include Perceived Ease of Use (PEOU) in the structural model. This decision was based on theoretical parsimony rather than empirical evidence of low variance: the key contribution of this study is the introduction of CMCP as a content-level antecedent within the TAM framework, and retaining a parsimonious base model (CMCP → PU → BI → LE) enables this hypothesized pathway to be tested with greater clarity within a SEM design. The exclusion of PEOU is acknowledged as a limitation in Section 4.6. Although TAM has been extensively used in research on online learning and educational technologies (Scherer et al., 2019), the original model emphasizes functional perceptions of technology. It lacks content-level determinants, hence constraining its explanatory power in terms of learning behaviors on content-based platforms like YouTube. Though TAM has since been expanded into more sophisticated frameworks, including TAM2, TAM3, UTAUT, and UTAUT2, the other constructs introduced in those frameworks (e.g., social influence, facilitating conditions, habit, and price value) were theorized mainly in organizational or commercial contexts of technology adoption (Venkatesh et al., 2003; Venkatesh et al., 2012). The target platform in this study is free, familiar to Thai adolescents, and voluntarily used to learn informally, making some of these constructs less relevant. Furthermore, the key theoretical contribution of the study is the addition of a content-level variable, Chinese Media Content Preference (CMCP) to the TAM framework; keeping a parsimonious base model enables the hypothesized CMCP → PU → BI path to be tested with greater clarity and statistical accuracy within a Structural Equation Modeling (SEM) design.

Uses and Gratifications Theory (UGT). UGT, proposed by Katz et al. (1973), is a communication studies approach to media use that theorizes the audience as an active consumer of media rather than a passive one. Its fundamental premise is that people actively choose and consume media content to meet their social and psychological needs and gain gratification. The gratification dimension in the UGT framework used in the present study is operationalized through the content preferences of Thai Chinese-language learners, specifically their willingness to engage with Chinese songs, movies and TV dramas, and variety shows on YouTube. These preferences are understood as a behaviorally based indicator of content-consumption gratification tendencies rather than a direct measure of affective states such as enjoyment, immersion, or flow. It is worth mentioning that UGT includes a wide range of gratifications, such as those obtained through social interaction options like commenting, sharing, and subscribing. The present study, however, is limited to content-consumption gratifications, i.e., cognitive and affective gratification gained by the learner due to engagement with specific forms of Chinese media content. The scope of the present study does not include social-interactive gratifications since CMCP is operationalized at the level of content-type preference and not platform-mediated social behavior. Notably, in informal language learning, long-term exposure to entertainment-focused target-language media may serve as a constant stream of input that facilitates incidental learning, and content preferences can be regarded as a theoretically relevant source of learning-oriented platform use.

Integration logic and research model. This study combines the content-gratification perspective of UGT with the technology-acceptance mechanism of TAM by introducing Chinese Media Content Preference as an external variable to expand the TAM. CMCP is operationalized as learners’ category-specific content preferences for Chinese entertainment media (songs, dramas, variety shows, etc.), serving as a narrow indicator of the active audience selection construct of UGT. CMCP is conceptualized as a multi-dimensional measure of preference strength of learners in significant categories of Chinese entertainment media on YouTube, such as Chinese songs, television dramas, variety, and reality shows. Every dimension is an indicator of a different source of content gratification; item-level measurement details are given in Section 2 (Method). This study suggests a partial-mediation framework whereby CMCP has a direct effect on BI (gratification-driven use) and an indirect effect through PU (instrumental valuation). The integrated model involves four fundamental pathways: (1) CMCP → PU; (2) CMCP → BI; (3) PU → BI; and (4) BI → LE. In this study, LE is characterized as self-perceived learning effectiveness, i.e., the subjective evaluation of improvements resulting from YouTube-based Chinese learning (e.g., perceived gains in listening comprehension, vocabulary knowledge, and cultural familiarity). Self-report is chosen as a pragmatic and widely utilized measure in non-formal learning settings where no standardized measures exist; therefore, the construct is specifically modeled as perceived learning effectiveness (cf. Benson, 2011).

Despite the extensive use of TAM in educational technology research since Davis (1989), the existing evidence base remains constrained when TAM is employed to explain informal, content-based learning on open video platforms like YouTube. Such limitations leave space to both theoretical development and empirical investigation in this study.

First, most TAM-based studies in the education sector have focused on formal learning technologies, including learning management systems, MOOCs, and mobile learning apps, in which learning processes are institutionalized and the technology is primarily intended to teach (Scherer et al., 2019; Granić and Marangunić, 2019). In comparison, YouTube is a user-generated content platform whereby the process of learning is usually self-initiated, loosely organized, and frequently combined with entertainment consumption. Although there has been some research on YouTube-related learning behaviors in higher-education contexts (e.g., Chintalapati and Daruri, 2017), empirical research on the use of TAM in informal language learning through YouTube is comparatively limited, particularly for Chinese as a foreign language and adolescent learners in Southeast Asia. The emphasis on secondary-school adolescents is especially justified since this group represents the main target of the institutionalized Chinese-language curriculum in Thailand (Bureau of International Cooperation, Ministry of Education of Thailand, 2023) and possesses almost universal internet coverage (National Statistical Office of Thailand, 2023) and high levels of penetration in YouTube (Jayuphan et al., 2026), but has been hardly addressed empirically in the context of YouTube-based language-learning studies.

Second, traditional TAM emphasizes functional perceptions of technology, with PU being a key BI driver. Nevertheless, on content-saturated platforms like YouTube, differences in learning experiences are not only influenced by the platform but also, more directly, by what learners choose to watch. That is, content preferences might be reasonable precursors to PU, as they influence whether YouTube is viewed as an effective learning resource in the first place. However, determinants at the level of content are hardly ever explicitly modeled in TAM-based research on YouTube learning, which poses an open empirical question about how content preferences are transformed into PU and further continued intention and learning outcomes. As mentioned above, PEOU was excluded from the model because of theoretical parsimony and to focus on the content-level pathway; its potential role should be examined in future research.

Third, UGT offers a complementary explanation of purposive media selection by theorizing users as active agents who select content to fulfill specific needs (Katz et al., 1973). This view is especially applicable to informal language learning on YouTube, where learners can access instructional or entertainment-oriented Chinese media (e.g., songs, dramas, variety shows) for various satisfactions. Although TAM and UGT have been combined in adjacent fields such as online streaming and digital consumption (Camilleri and Falzon, 2021), learner-centered modeling research that applies an integrated TAM-UGT lens to YouTube-based language learning, particularly in a Chinese-as-a-foreign-language setting, remains limited.

Fourth, in the YouTube language-learning literature, instructional videos (e.g., grammar explanations and vocabulary tutorials) have been studied more frequently, whereas entertainment-oriented target-language content that learners watch extensively in daily life has received less attention. For Thai adolescent learners, watching Chinese-language entertainment content is a convenient way to receive sustained exposure to the target language and may lead to incidental learning and familiarity with the target culture. However, little research has examined whether and how preferences for entertainment-oriented Chinese media lead to effective learning via a chain of mechanisms involving PU and BI.

To fill these gaps, this study incorporates TAM and UGT to model the informal Chinese learning of Thai adolescents on YouTube. It introduces a content-preference variable, CMCP, which grounded in the gratification-seeking rationale of UGT. It uses SEM to test a mechanism pathway between CMCP and PU, BI, and LE, thereby linking learners’ content preferences to their assessment of the value of learning through YouTube and the results associated with long-term engagement.

Based on the theoretical framework and research gaps outlined above, the present study addresses the following research questions:

*RQ1*: To what extent does CMCP predict Thai adolescents’ PU of YouTube for Chinese-language learning?

*RQ2*: To what extent does CMCP predict BI, and is this association mediated by PU (i.e., a partial-mediation structure with both direct and indirect effects)?

*RQ3*: To what extent does BI predict self-perceived LE?

These questions are examined using SEM to estimate both direct and indirect effects.

Based on the integrated TAM–UGT theoretical framework and the research questions above, the present study proposes the following hypotheses:

*H1*: CMCP positively predicts PU of YouTube for Chinese-language learning.

*H2*: PU positively predicts BI to continue using YouTube for informal Chinese learning.

*H3*: CMCP positively predicts BI (direct effect).

*H4*: BI positively predicts LE.

*Indirect-effect test (not formulated as a separate hypothesis).* In line with the proposed partial-mediation structure, the study additionally estimates the indirect effect of CMCP on BI via PU (CMCP → PU → BI) using SEM with bootstrapped confidence intervals.

Theoretical contributions. The study contributes to the study of informal, platform-based language learning by incorporating TAM and UGT in a model of Thai adolescents using YouTube to learn Chinese (CMCP → PU → BI → LE). First, it expands TAM studies to less formal educational technologies, namely content-driven and user-generated video environments, thereby adding evidence on how TAM mechanisms operate in less structured learning environments (Granić and Marangunić, 2019). Second, it integrates a content-gratification approach by introducing CMCP as a theoretically driven antecedent, connecting learners’ choice of content (what they watch) with their perceived instrumental value of the platform (how useful they find it). Third, it expands the model’s explanatory power beyond continued intention to self-perceived learning effectiveness, providing a bridge between technology-related perceptions and perceived learning gains in informal learning settings.

Practical contributions. The results have implications for Chinese-language teachers, YouTubers and other educational stakeholders. Contribute to educators by recognizing the viewership preference categories of Chinese media among Thai adolescents and explaining how these preferences are associated with PU and continuous use intention, which will assist in effectively integrating entertainment-focused Chinese videos into both classroom and out-of-class learning activities. As for content creators, the model suggests that the retention of engagement is co-determined by content preference and PU, while offering evidence-based guidance about how to produce attractive as well as input-affiliated (e.g., clearer language scaffolding, subtitle support, level-appropriate input) videos. For policymakers and program designers, the findings offer preliminary evidence, based on two participating schools, on how popular video platforms could be leveraged for enhancing long-term engagement and perceived learning outcomes. However, broader policy recommendations would require validation with more diverse and representative samples across Thailand.

## 2 Materials and methods

### 2.1 Study design

A cross-sectional survey design was employed for this study. Specifically, the study focused on understanding how CMCP, PU, BI, and self-perceived LE were interrelated in the informal use of YouTube among Thai secondary school students learning Chinese. Data were collected with an anonymous online questionnaire implemented in Wenjuanxing^1^ from Aug 2 to 13, 2024. The questionnaire was distributed to the secondary schools of Chonprathan Wittaya School in Nonthaburi Province and Chanthaburi Federation School in Chanthaburi Province, Thailand. The Chonprathan Wittaya School is a private school located in the Bangkok metropolitan area, catering to mostly urban middle-class students. Chanthaburi Federation School is a public school located in a rural area of Chanthaburi Province, with comparatively fewer educational resources.

### 2.2 Participants and sampling

The study involved 346 secondary school students. The purposive sampling was used with the following inclusion criteria: (a) enrolled in grades 7 to 12 (Mathayom 1 to 6) at either of the two participating schools, (b) currently studying Chinese as a school subject, and (c) having access to YouTube. There were no other exclusion criteria during the recruitment process. Rather, data quality screening was conducted post-collection (see Section 2.4).

The screening process yielded 266 valid questionnaires, which were retained for analysis, resulting in an effective response rate of 76.88%. The male (59.77%) and female (40.23%) respondents were among the valid ones. The participants’ ages ranged from 13 to 19 years, with most aged 13–14 years (43.23%). The highest percentage (33.83%) was grade 9 students, followed by grade 7 (31.20%) and grade 12 (23.68%). The majority of the participants (60.15%) had studied Chinese for more than 2 years. Over 90% (90.60%) did not take the HSK exam, and 40.60% reported being of ethnic Chinese origin.

For the minimum sample size, an *a priori* power analysis was conducted using G*Power 3.1 (Faul et al., 2009). It was assumed that the model to be analyzed is a linear multiple regression with two predictors, equivalent to the maximum number of predictors for any one endogenous variable in the structural model. The minimal sample size needed was 68 with a medium effect size (*f*^2^ = 0.15), *α* = 0.05, and a statistical power of 0.80. This threshold was significantly surpassed by the final sample of 266. The sample also met the typical recommended minimum of 200 cases for SEM using maximum likelihood estimation (Kline, 2017).

### 2.3 Procedure

The study was conducted during the teaching practicum in Thailand by the researcher from June to August 2024, which was organized by the undergraduate university of the researcher in China. The researcher had contacted Chinese-language teachers at both schools before data collection. The survey distribution was also aided by the teachers as part of their routine school activities.

The teachers distributed the questionnaire QR code to the students through social media (WeChat and LINE) outside of the classroom. The survey was done by students individually on their personal devices. To meet different degrees of Chinese proficiency among the respondents, the questionnaire was given in a bilingual format between Chinese and Thai. It was voluntary and anonymous. No personally identifiable information was obtained, and no incentives were offered. Each questionnaire required about 5–10 min to complete.

### 2.4 Data quality control

To maintain data quality, the study applied two screening criteria. First, responses with an average completion time of fewer than 100 s based on questionnaire length were removed. The survey included 30 questions (24 Likert-type scale items and 6 demographic questions). This threshold was guided by median-based speeder criteria in the study of Greszki et al. (2015), who advised marking responses that were 30, 40%, or 50% faster than the median completion time. The median completion time observed in this study was 169 s, and the 100-s threshold equates to about 41% greater speed than the median; thus, it is consistent with the previously mentioned 40% criterion. This criterion rejected 66 responses. Second, straight-lining was defined as choosing the same response option for at least 90% of the 24 Likert-type scale items (6 items were demographics and not included). This pattern was unconfounded responding, and led to the exclusion of a further 13 responses. This left a total of 266 valid questionnaires (effective response rate = 76.88%) after the screening process. In the retained sample, there was no missing data, as submitting each question was mandatory on the online platform.

### 2.5 Measures

All constructs were measured on a 5-point Likert scale. For CMCP and BI, response options ranged from 1 (very unwilling) to 5 (very willing). For PU and LE, response options ranged from 1 (strongly disagree) to 5 (strongly agree).

The items were initially written in Chinese and then translated into Thai by a bilingual teacher of the Chinese language at the school that participated. A second bilingual teacher at the same school independently reviewed the translation for semantic accuracy and cultural appropriateness. Some minor wording adjustments were made based on the review before administration. The final questionnaire was presented in both Chinese and Thai languages. In the Results section, reliability and validity indices of all scales are reported.

#### 2.5.1 Chinese media content preference (CMCP)

The construct was developed for this study within the framework of the Uses and Gratifications Theory (Katz et al., 1973). According to the theory, media users are active in choosing content to fulfill certain needs. Three questions measured the readiness of participants to consume Chinese-language entertainment content on YouTube within three types of media: Chinese songs (Q13), Chinese movies and TV dramas (Q14), and Chinese variety shows (Q15). One of the sample items is Are you willing to listen to Chinese songs on YouTube?

#### 2.5.2 Perceived usefulness (PU)

This construct was developed based on the PU dimension of TAM (Davis, 1989). Three questions were used to assess how useful participants found YouTube as a tool for learning Chinese. These items covered perceived learning efficiency (Q20), perceived improvement in Chinese grades (Q21), and perceived ability to meet learning needs (Q23). An example of such an item is: I believe that it can be more efficient to learn Chinese with the help of YouTube.

#### 2.5.3 Behavioral intention (BI)

The construct was adapted from behavioral intention measures commonly used in TAM studies (Davis, 1989; Venkatesh et al., 2003). Five items were used to assess participants’ willingness to use YouTube to learn Chinese. These items included willingness to use YouTube for learning Chinese (Q8), willingness to continue using it in the future (Q10), willingness to recommend it to others (Q11), willingness to watch specific Chinese teaching videos (Q12), and willingness to learn Chinese on YouTube every day (Q16). An example item is Do you intend to keep watching YouTube videos to learn Chinese in the future?

#### 2.5.4 Self-perceived learning effectiveness (LE)

This construct was developed for this study to measure learners’ self-perceived language gains from using YouTube-based Chinese learning. Four items measured perceived improvement in major areas of language skills: vocabulary development (Q26), oral fluency (Q27), character writing skills (Q28), and listening comprehension (Q29). One example is that I have more vocabulary after watching Chinese videos on YouTube.

### 2.6 Ethics statement

The Office of Scientific Research, School of Chinese Language and Culture, Zhejiang Yuexiu University, approved this study and provided ethics exemption. The research was identified as a low-risk, non-interventional, anonymous social science survey.

Before the questionnaire was completed, all participants were informed of the research purpose, the principle of voluntary participation, and their right to withdraw at any stage without consequence. All students provided informed assent by indicating their willingness to participate before proceeding to the questionnaire items.

Since all respondents were minors (13–19 years old), the questionnaire was structured and distributed by school teachers as part of a regular school activity. The distribution was done by the teachers; students filled out the survey individually after school. Since the survey is anonymous, no interventional measures were taken, and there was minimal risk to the participants, the institutional ethics review waived the need for separate written parental informed consent. Data were stored in password-protected machines that only the research team could access and use in academic research activities.

### 2.7 Data analysis

SPSS 27.0 was used to carry out descriptive statistics and bivariate correlation analyses. AMOS 27.0 with Maximum Likelihood (ML) estimation was used to perform Confirmatory Factor Analysis (CFA) and SEM.

The analysis was done in the following ways. First, the normality of all observed variables was tested using skewness and kurtosis values. The criteria of |skewness| < 2 and |kurtosis| < 7 were used (Kline, 2017). Second, a four-factor CFA measurement model was estimated to assess model fit, factor loadings, construct reliability (Cronbach’s *α* and composite reliability), and convergent validity (average variance extracted). Fornell-Larcker criterion (Fornell and Larcker, 1981) and heterotrait-monotrait (HTMT) ratio (Henseler et al., 2015) were used to determine discriminant validity. Model fit was assessed with reference to the following: *χ*^2^/*df* < 3, CFI > 0.90, TLI > 0.90, RMSEA < 0.08, and SRMR < 0.08 (Hu and Bentler, 1999; Hair et al., 2018).

Third, the structural model was estimated to determine the hypothesized paths: CMCP → PU (H1), PU → BI (H2), CMCP → BI (H3), and BI → LE (H4). The reported values include standardized path coefficients, standard errors, and significance levels. Fourth, the indirect influence of CMCP on BI by means of PU was also tested with the bootstrap technique with 5,000 resamples and 95% bias-corrected confidence intervals. Fifth, the presence of common method bias was determined through comparison of a single-factor CFA model fit and four-factor measurement model (Podsakoff et al., 2003). Additionally, the full collinearity VIF approach (Kock, 2015) was conducted using R (version 4.5.3; R Core Team, 2026). An alternative three-factor CFA model, in which PU and BI were combined into a single factor, was also estimated to further assess discriminant validity between these two constructs. Lastly, hierarchical regression analyses were done as robustness tests to confirm that the results were consistent with the SEM findings.

## 3 Results

### 3.1 Normality assessment

Before the main analysis, the univariate normality of all 15 observed variables was examined. Skewness values ranged from −1.062 to −0.008, and kurtosis values ranged from −1.129 to 0.295. All values fell within the recommended thresholds of |skewness| < 2 and |kurtosis| < 7 (Kline, 2017), indicating that the data were approximately normally distributed. Maximum likelihood estimation was therefore considered appropriate.

### 3.2 Descriptive statistics and correlation analysis

Table 1 summarizes the sample characteristics and descriptive statistics, as well as the bivariate correlations among the four latent variables. Means ranged from 3.37 (LE) to 3.83 (CMCP), and standard deviations ranged from 0.94 (BI) to 1.10 (CMCP). All four constructs were significantly and positively correlated (*p* < 0.01). The strongest correlation was between PU and LE (*r* = 0.74, *p* < 0.01), followed by PU and BI (*r* = 0.71, *p* < 0.01). The weakest correlation was between CMCP and PU (*r* = 0.48, *p* < 0.01). Cronbach’s *α* coefficients were between 0.83 and 0.91, all larger than 0.70.

**Table 1:** Sample characteristics, descriptive statistics, and correlations among latent variables.

A: Sample characteristics			
Variable	Category	*n*	%
Gender	Male	159	59.77
	Female	107	40.23
Age	13–14	115	43.23
	14–15	77	28.95
	16–17	52	19.55
	18–19	22	8.27
Grade	Grade 7	83	31.20
	Grade 8	18	6.77
	Grade 9	90	33.83
	Grade 10	1	0.38
	Grade 11	11	4.14
	Grade 12	63	23.68
Chinese learning duration	< 6 months	76	28.57
	6–12 months	10	3.76
	1–2 years	20	7.52
	> 2 years	160	60.15
HSK level	HSK 1	13	4.89
	HSK 2	6	2.26
	HSK 3	4	1.50
	HSK 4	2	0.75
	Not taken	241	90.60
Ethnic Chinese heritage	Yes	108	40.60

B: Descriptive statistics and correlations						
Variable	*M*	*SD*	1	2	3	4
1. Chinese Media Content Preference (CMCP)	3.83	1.10	(0.83)			
2. Perceived Usefulness (PU)	3.60	0.96	0.48**	(0.83)		
3. Behavioral Intention (BI)	3.70	0.94	0.65**	0.71**	(0.88)	
4. Self-perceived Learning Effectiveness (LE)	3.37	1.07	0.49**	0.74**	0.66**	(0.91)

*N* = 266. Values in parentheses on the diagonal are Cronbach’s α coefficients. M, Mean; SD, Standard deviation. Age was assessed using pre-defined questionnaire categories; respondents selected one category that best matched their age. *p < 0.05, **p < 0.01, ***p < 0.001.

### 3.3 Measurement model

Confirmatory factor analysis (CFA) was conducted to evaluate the four-factor measurement model. The model fit indices were as follows: *χ*^2^(84) = 175.673 (*p* < 0.001), *χ*^2^/*df* = 2.091, GFI = 0.921, CFI = 0.966, TLI = 0.957, NFI = 0.937, IFI = 0.966, RMSEA = 0.064 (90% CI [0.051, 0.077]), SRMR = 0.042. All indices met the recommended thresholds (Hu and Bentler, 1999; Hair et al., 2018), confirming good model fit.

Table 2 summarizes the factor loadings, reliability, and convergent validity results. Standardized factor loadings ranged from 0.632 to 0.927, all exceeding the 0.50 threshold and significant at *p* < 0.001. Cronbach’s *α* values ranged from 0.826 to 0.910, and composite reliability (CR) values ranged from 0.831 to 0.912, all exceeding 0.70. The average variance extracted (AVE) ranges from 0.611 to 0.723, exceeding the recommended benchmark of 0.50 (Fornell and Larcker, 1981). These findings indicate satisfactory internal consistency reliability and convergent validity.

**Table 2:** Measurement model results: Factor loadings, reliability, and convergent validity.

Construct	Item	Loading	*α*	CR	AVE
Chinese Media Content Preference (CMCP)	Q13 Chinese songs	0.632	0.826	0.831	0.627
	Q14 Chinese movies/dramas	0.810			
	Q15 Chinese variety shows	0.908			
Perceived Usefulness (PU)	Q20 Improve learning efficiency	0.795	0.831	0.833	0.624
	Q21 Improve Chinese grades	0.801			
	Q23 Meet learning needs	0.773			
Behavioral Intention (BI)	Q12 Chinese teaching videos	0.830	0.875	0.886	0.611
	Q8 Willing to use YouTube for learning	0.818			
	Q10 Continue using in the future	0.812			
	Q11 Recommend to others	0.781			
	Q16 Daily learning intention	0.653			
Self-perceived Learning Effectiveness (LE)	Q26 Vocabulary improvement	0.816	0.910	0.912	0.723
	Q27 Speaking fluency improvement	0.927			
	Q28 Writing ability improvement	0.807			
	Q29 Listening comprehension improvement	0.845			

*N* = 266. Loading, standardized factor loading; α, Cronbach’s alpha; CR, composite reliability; AVE, average variance extracted. All factor loadings are significant at *p* < 0.001. Recommended thresholds: Loading > 0.50, α > 0.70, CR > 0.70, AVE > 0.50 (Hair et al., 2018).

### 3.4 Discriminant validity

Table 3 shows the results of discriminant validity based on the Fornell-Larcker criterion (Fornell and Larcker, 1981) and the heterotrait-monotrait (HTMT) ratio (Henseler et al., 2015). The off-diagonal values are correlations between constructs as estimated by the CFA measurement model, so these do not match observed Pearson correlations of composite scores presented in Table 1. The square root of AVE values were 0.792 (CMCP), 0.790 (PU), 0.781 (BI), and 0.850 (LE), all exceeding the corresponding off-diagonal correlations (ranging from 0.484 to 0.739). The full HTMT matrix is presented in Table 3 (Panel B). All HTMT values were below the strict 0.85 threshold except PU-LE (0.852), which remained below the more lenient 0.90 criterion (Henseler et al., 2015; Hair et al., 2018). Notably, the PU-BI HTMT value was 0.827, below the 0.85 threshold, supporting the discriminant validity between these two constructs that is central to the mediation analysis. Thus, overall discriminant validity was acceptable. As an additional test of discriminant validity between PU and BI, an alternative three-factor model in which PU and BI items were loaded onto a single factor was estimated. The three-factor model demonstrated significantly poorer fit (*χ*^2^ = 280.076, *df* = 87, CFI = 0.928, RMSEA = 0.091) than the four-factor model (Δ*χ*^2^ = 104.4, Δ*df* = 3, *p* < 0.001), confirming that PU and BI are empirically distinct constructs.

**Table 3:** Discriminant validity: Fornell-Larcker criterion and HTMT ratios.

Variable	1	2	3	4
A: Fornell-Larcker Criterion				
1. CMCP	**0.792**			
2. PU	0.484	**0.790**		
3. BI	0.648	0.709	**0.781**	
4. LE	0.488	0.739	0.663	**0.850**
B: HTMT ratios				
1. CMCP	—			
2. PU	0.581	—		
3. BI	0.752	0.827	—	
4. LE	0.564	0.852	0.738	—

*N* = 266. A: Diagonal values in bold represent the square root of AVE for each construct. Off-diagonal values represent inter-construct correlations estimated from the CFA measurement model. Discriminant validity is established when the square root of AVE for each construct exceeds its correlations with other constructs (Fornell and Larcker, 1981). B: HTMT = heterotrait-monotrait ratio (Henseler et al., 2015). All HTMT values were below the strict 0.85 threshold except PU-LE (0.852), which remained below the more lenient 0.90 criterion. The PU-BI HTMT value was 0.827, supporting the discriminant validity central to the mediation analysis.

### 3.5 Common method bias

Since all data were collected through self-report questionnaires, the common method bias (CMB) was examined. A single-factor CFA model, in which all 15 measurement items were constrained to load onto a single factor, was compared with the four-factor measurement model. The single-factor model demonstrated significantly poorer fit (*χ*^2^ = 655.193, *df* = 90, CFI = 0.789, RMSEA = 0.154) than the four-factor model (*χ*^2^ = 175.673, *df* = 84, CFI = 0.966, RMSEA = 0.064). The difference in CFI (ΔCFI = 0.177) far exceeded the 0.01 threshold (Cheung and Rensvold, 2002), and the chi-square difference was statistically significant (Δ*χ*^2^ = 479.52, Δ*df* = 6, *p* < 0.001). These results suggest that CMB did not pose a serious threat to the validity of the findings (Podsakoff et al., 2003). Additionally, the full collinearity variance inflation factor (VIF) approach recommended by Kock (2015) was applied as a supplementary test. All VIF values were below the 3.3 threshold (CMCP = 1.743, PU = 2.716, BI = 2.788, LE = 2.433), suggesting that CMB was unlikely to fully account for the observed relationships.

### 3.6 Structural model and hypothesis testing

After confirming the measurement model, the structural model was tested. The model fit indices were as follows: *χ*^2^/*df* = 2.672, CFI = 0.946, TLI = 0.934, RMSEA = 0.079, SRMR = 0.059, NFI = 0.917, IFI = 0.947. GFI was 0.898, which is less than the ideal 0.90 (Hair et al., 2018) but more than the acceptable level of 0.80. The model fit overall was considered adequate.

**Figure 1:**
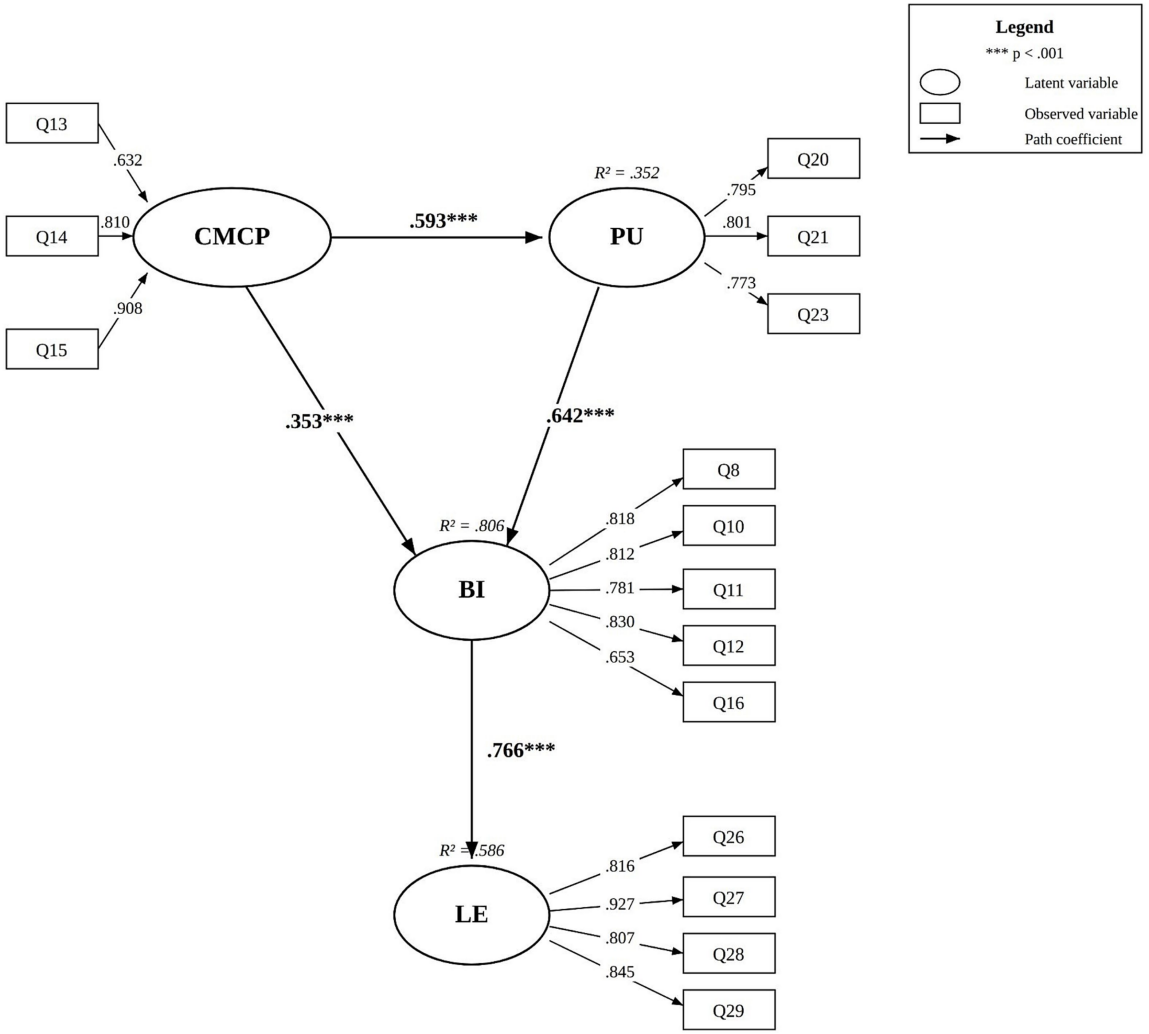
Structural model with standardized path coefficients.

Table 4 summarizes the structural path coefficients and hypothesis testing results. All four hypothesized paths were significant at *p* < 0.001. CMCP positively predicted PU (*β* = 0.593, SE = 0.098, *z* = 7.362, *p* < 0.001), supporting H1. This path accounted for 35.2% of PU variance (R^2^ = 0.352). PU positively predicted BI (*β* = 0.642, SE = 0.070, *z* = 9.241, *p* < 0.001), supporting H2. CMCP had a direct positive effect on BI (*β* = 0.353, SE = 0.079, *z* = 5.515, *p* < 0.001), supporting H3. CMCP and PU jointly explained 80.6% of the variance in BI (R^2^ = 0.806). BI positively predicted LE (*β* = 0.766, SE = 0.068, *z* = 11.618, *p* < 0.001), supporting H4. This path explained 58.6% of the variance in LE (R^2^ = 0.586). Figure 1 illustrates the structural model with standardized path coefficients.

**Table 4:** Structural model results: path coefficients and hypothesis testing.

A: Structural path results						
Hypothesis	*β*	*SE*	*Z(CR)*	*p*	*R* ^2^	Result
H1: CMCP → PU	0.593	0.098	7.362	<0.001	0.352	Supported
H2: PU → BI	0.642	0.070	9.241	<0.001		Supported
H3: CMCP → BI	0.353	0.079	5.515	<0.001	0.806	Supported
H4: BI → LE	0.766	0.068	11.618	<0.001	0.586	Supported

B: Model fit indices	
Index	Value
*χ*^2^/*df*	2.672
GFI	0.898
RMSEA	0.079
RMR	0.081
SRMR	0.059
CFI	0.946
NFI	0.917
TLI	0.934
IFI	0.947

*N* = 266. *β*, standardized path coefficient; SE, standard error; CMCP, Chinese Media Content Preference; PU, Perceived Usefulness; BI, Behavioral Intention; LE, self-perceived Learning Effectiveness. Model fit criteria: χ^2^/df < 3, CFI > 0.90, TLI > 0.90, RMSEA < 0.08, SRMR < 0.08, GFI > 0.90 (ideal)/>0.80 (acceptable) (Hu and Bentler, 1999; Hair et al., 2018). *p < 0.05, **p < 0.01, ***p < 0.001.

### 3.7 Mediation analysis

Table 5 presents the results of the mediation analysis. The bootstrap method with 5,000 resamples was used to test the mediating role of PU between CMCP and BI. The total effect of CMCP on BI was 0.553 (SE = 0.037, 95% CI [0.480, 0.625]). After controlling for the mediator, the direct effect was 0.142 (SE = 0.038, 95% CI [0.068, 0.217]), which remained significant. The indirect effect through PU was 0.410 (SE = 0.050, 95% CI [0.318, 0.511]). The 95% bootstrap confidence interval did not include zero, confirming the significance of the mediation effect. The indirect effect accounted for 74.22% of the total effect, while the direct effect accounted for 25.78%. Because both the direct and indirect effects were significant, PU partially mediated the CMCP-to-BI relationship.

**Table 5:** Mediation effect analysis results with bootstrap 95% confidence intervals.

Effect	Estimate	*SE*	95% CI	Ratio	Conclusion
Total effect (c): CMCP → BI	0.553**	0.037	[0.480, 0.625]	—	—
Direct effect (c’): CMCP → BI	0.142**	0.038	[0.068, 0.217]	25.78%	Significant
Indirect effect (a × b): CMCP → PU → BI	0.410**	0.050	[0.318, 0.511]	74.22%	Partial mediation
Path a: CMCP → PU	0.567**	0.035	[0.497, 0.636]	—	—
Path b: PU → BI	0.724**	0.047	[0.632, 0.816]	—	—

*N* = 266. Bootstrap samples = 5,000. CI, confidence interval; SE, bootstrap standard error. Mediation is significant when the 95% CI does not include zero. Ratio = effect/total effect. Ratios were calculated using unrounded estimates. *p < 0.05, **p < 0.01, ***p < 0.001.

The bootstrapping of observed composite scores was used to perform mediation analysis and is presented as unstandardized effects. These values may vary slightly from the standardized SEM path coefficients provided in Table 4. All directions of paths and their significance levels were in accordance across both approaches.

### 3.8 Robustness checks

Hierarchical regression analyses were conducted to verify the robustness of the SEM results. In the first model, BI was regressed on CMCP in Step 1. The model was significant (R^2^ = 0.459, *F*(1, 264) = 224.281, *p* < 0.001). In Step 2, PU was added, resulting in a significant increment in explained variance (ΔR^2^ = 0.258, ΔF(1, 263) = 240.018, *p* < 0.001). The standardized coefficient for PU was *β* = 0.715 (*p* < 0.001). In the second model, BI significantly predicted LE (*β* = 0.735, *p* < 0.001), accounting for 54.1% of the variance. The direction and significance of all regression coefficients were consistent with the SEM path estimates, confirming the robustness of the findings.

## 4 Discussion

The study integrated the TAM and UGT to examine Thai secondary school students’ informal Chinese-language learning using YouTube. The results supported all four of the proposed paths, and a mediation analysis confirmed that perceived usefulness partially mediated the effect of Chinese media content preferences on behavioral intention. The results in relation to each hypothesis, commented on theoretical and practical implications of such findings, as well as limitations of the research, are discussed in the following subsections.

### 4.1 Discussion of hypothesis testing results

H1 hypothesized that CMCP is a positive predictor of PU. This hypothesis was confirmed (*β* = 0.593, *p* < 0.001), and CMCP accounted for 35.2% of the variance in PU. The result implies that the stronger students are in their preference for Chinese entertainment media, such as songs, movies, dramas, and variety shows, the more likely they are to perceive YouTube as a useful tool for learning Chinese. This outcome aligns with the fundamental assumption of UGT, which assumes that people actively choose media content that meets their needs and derive gratification from such use (Katz et al., 1973). It should be mentioned that the CMCP items reflected the willingness of learners to interact with certain types of media instead of affective states per se. Nevertheless, motivationally, this expressed willingness can be seen as an indication of underlying intrinsic interest and enjoyment. Although not directly measured in this study, it is possible that learners who prefer Chinese entertainment media may experience flow-like states of engagement (Csikszentmihalyi, 1990), which could help sustain attention and facilitate deeper processing of language input. Future research could incorporate direct measures of flow and enjoyment to empirically examine this possibility. This finding builds on previous TAM literature by showing that content-level gratification may be used as a valuable external variable predicting PU in informal learning settings, outside the technology-focused variables usually studied in TAM research (Davis, 1989; Scherer et al., 2019).

H2 hypothesized that BI is positively predicted by PU. This hypothesis was confirmed (*β* = 0.642, *p* < 0.001). It was the second-largest path coefficient in the structural model. The result aligns with a large body of TAM research showing that PU can be used as a strong predictor of BI in educational technology settings (Davis, 1989; Venkatesh and Davis, 2000; Scherer et al., 2019). It confirms that Thai adolescents are more likely to use YouTube again when they find it helpful in enhancing their learning efficiency, improving their grades, and meeting their learning needs. The finding also suggests that the TAM framework could be applied to informal, platform-based learning contexts.

H3 hypothesized that CMCP has a positive direct impact on BI, independent of PU. This hypothesis was confirmed (*β* = 0.353, *p* < 0.001). This result indicates that the preferences of Thai adolescents toward Chinese entertainment content not only increase perceived usefulness but also directly stimulate their intention to use YouTube to learn Chinese. According to UGT, this direct path reflects the intrinsic gratification students derive from consuming enjoyable Chinese media content (Katz et al., 1973). The intrinsic motivation can be interpreted as a direct effect: if learners perceive the content as enjoyable, they will be motivated to proceed with it despite any rational evaluation of its instrumental value (Ryan and Deci, 2000). The enjoyment and satisfaction derived from Chinese songs, dramas, and variety shows, even when perceived usefulness is kept constant, are independent factors that make learners continue using the platform. This dual-pathway result (CMCP to BI both directly and indirectly via PU) gives empirical evidence to the combination of TAM and UGT and suggests that the content gratification has a unique role, which cannot be entirely explained by the usefulness construct.

H4 hypothesized that BI has a positive effect on LE. This hypothesis was confirmed (*β* = 0.766, *p* < 0.001), which was the strongest path in the model. BI accounted for 58.6% of the variance in LE. The result implies that higher behavioral intention to use YouTube to learn Chinese was associated with greater self-perceived learning achievements in vocabulary, speaking, writing, and listening. The robustness of the BI → LE relationship is in line with the larger body of literature on self-regulated learning, which indicates that those learners who are more purposeful about their learning experiences tend to report improved perceived outcomes (Zimmerman, 2002). It also aligns with prior research on informal language learning, which has shown that prolonged voluntary exposure to target-language media can support vocabulary acquisition and listening comprehension (Arndt and Woore, 2018; Peters and Webb, 2018). In the present study, however, the outcome refers to self-perceived rather than objectively assessed gains.

From a second language acquisition (SLA) perspective, the BI → LE pathway can be contextualized through the lens of Krashen’s (1985) input hypothesis, which posits that language development is facilitated when learners are exposed to comprehensible input slightly above their current proficiency level. YouTube can provide a rich source of such input through Chinese entertainment media, where visual context, subtitles, and familiar narrative structures can scaffold comprehension. Furthermore, the concept of incidental learning (Hulstijn, 2001) suggests that learners can acquire vocabulary and grammatical knowledge as a by-product of meaning-focused engagement with target-language content, without explicit instructional intent. The sustained voluntary engagement reflected in higher BI scores may thus create favorable conditions for incidental learning, which in turn is reflected in stronger self-perceived learning effectiveness. However, as noted above, the present study measured perceived rather than objectively assessed gains, and future research should triangulate self-reports with standardized proficiency measures to examine whether these perceptual gains correspond to actual language development.

### 4.2 Discussion of mediation results

The mediation analysis indicated that the CMCP → BI relationship was partially mediated by PU, with an indirect effect (74.22%) being significantly higher than the direct effect (25.78%). These ratios were estimated based on unrounded bootstrap estimates. This result means that the main mechanism through which Chinese media content preference is associated with behavioral intention is through perceived usefulness. That is, when Thai adolescents like Chinese entertainment content, they hold more positive impressions of YouTube’s utility for language learning, which in turn leads them to intend to use the platform more.

According to the partial mediation pattern, both the cognitive evaluation pathway (via PU) and the direct gratification pathway contribute to behavioral intention. Nevertheless, the dominance of the indirect pathway highlights that perceived usefulness is the main transmission mechanism. Psychologically, the fact that the indirect effect (74.22%) is larger than the direct effect (25.78%) shows an important integration process between affect and cognition. Thai adolescents did not stay at the level of hedonic consumption of entertainment content. Instead, the pattern suggests that after affective engagement, there may be a more reflective, utility-focused assessment. This aligns with accounts of self-regulated learning where motivational entry points are consolidated by perceived instrumentality (Pintrich, 2000). The finding, therefore, helps understand how affect and cognition interact in technology-mediated informal learning better.

The integration of TAM and UGT in this study goes beyond simply adding a UGT-derived variable to a TAM model. The partial mediation pattern suggests how the affective and cognitive dimensions of the two theories may interact in a sequential process. UGT explains why learners actively select specific Chinese entertainment content on YouTube: they are drawn to particular media types based on their content preferences, which may reflect a gratification-seeking orientation (Katz et al., 1973). TAM, in turn, explains how this preference is cognitively translated into an instrumental evaluation: learners who prefer such content may be more likely to perceive the platform as useful for learning (PU), which then strengthens their intention to continue using it (BI). The dominance of the indirect pathway (74.22%) over the direct pathway (25.78%) suggests that this cognitive translation process is the primary mechanism, while the significant direct effect indicates that content preference also independently sustains engagement. This dual-pathway structure suggests that TAM and UGT are not merely complementary but may operate in a sequentially linked manner: content preference serves as the motivational entry point that feeds into the technology acceptance process. However, given the cross-sectional nature of the data, the directionality of this sequence remains to be confirmed by longitudinal research.

### 4.3 Discussion of the high explained variance in behavioral intention

The R^2^ value for BI was 0.806, indicating that CMCP and PU together accounted for 80.6% of the variance in behavioral intention. This is a comparatively high figure that deserves both theoretical and methodological attention.

The TAM framework posits that PU is a fundamental driver of BI (Davis, 1989), and a large body of research on TAM has shown that PU has high explanatory power for BI (Venkatesh and Davis, 2000). The sample used in the study comprised adolescent learners aged 13 to 19, for whom attitudes and behaviors related to technology use are likely to be more consistent (Teo, 2013). When adolescents find a learning tool useful, they are more willing to express an intention to use it, indicating a relatively close relationship between perceived instrumental value and stated intention. In addition, the complementary explanatory functions of CMCP (emotional motivation based on UGT) and PU (cognitive evaluation based on TAM) together explain a significant portion of variance in BI.

Methodologically, several factors may contribute to this high R^2^ value. The PU-BI observed correlation was *r* = 0.71, and their CFA-estimated correlation was 0.709. Although notable, these values are consistent with the typically strong PU-BI relationship reported in TAM research (Venkatesh and Bala, 2008). Importantly, the HTMT ratio for PU-BI was 0.827, below the strict 0.85 threshold, and a comparison between the four-factor CFA model and an alternative three-factor model in which PU and BI were merged showed significantly poorer fit for the latter (Δ*χ*^2^ = 104.4, Δ*df* = 3, *p* < 0.001), confirming that PU and BI are empirically distinct constructs despite sharing substantial variance. Also, the cross-sectional self-report design implies that common method variance may have increased the associations to some degree. However, the single-factor CFA comparison, the full collinearity VIF test (Kock, 2015), and the use of different scale anchors across constructs (willingness for CMCP and BI; agreement for PU and LE) as a procedural remedy (Podsakoff et al., 2003) collectively suggest that CMB was not a severe threat. Nevertheless, it should be acknowledged that all self-report, cross-sectional designs carry inherent residual CMB risk that cannot be fully eliminated through *post hoc* statistical tests alone (Podsakoff et al., 2003). Future research could mitigate this limitation by incorporating objective measures (e.g., platform usage logs) or multi-wave data collection designs.

### 4.4 Theoretical implications

This study contributes three theoretical aspects. First, it is an extension of TAM, adding Chinese Media Content Preference as a content-gratification variable based on UGT. Although previous studies of TAM in education have been mostly concentrated around formal learning technologies and technology-focused predictors (Scherer et al., 2019; Granić and Marangunić, 2019), this study shows that factors at the content-level may be used as meaningful external variables within the TAM framework, especially when it comes to informal learning settings where learners themselves choose media content.

Second, the study offers empirical support to the combination of TAM and UGT in describing the behavior of informal language learning. The dual-pathway result, whereby CMCP has direct and indirect effects on BI via PU, indicates that the two theories provide complementary explanatory power. UGT helps explain learners’ content-selection tendencies and gratification-seeking orientation, whereas TAM explains the cognitive appraisal of the instrumental value of the platform. This integration fills a gap in the literature as TAM and UGT have been scarcely used together to study content-driven learning on open video platforms (Camilleri and Falzon, 2021).

Third, the study generalizes the TAM-based evidence base to a new population (Thai secondary school students) and context (informal Chinese-language learning on YouTube). The majority of TAM research in language education has been conducted with adult learners or formal educational technologies. The results indicate that the TAM mechanism is effective among adolescent learners in an entertainment-oriented, self-directed learning environment.

### 4.5 Practical implications

The results have a number of practical implications for Chinese-language educators, content developers, and educational stakeholders. The following practical implications are derived from the current sample of secondary school students at two schools in Thailand and should be interpreted within this scope. To begin with, the strong positive correlation between CMCP and PU implies that Chinese-language teachers in Thailand can leverage students’ existing preference for Chinese entertainment media to increase perceived learning value. The incorporation of Chinese songs, dramas, and variety-show clips into the classroom teaching can serve as a bridge between informal and formal learning. In particular, teachers would be able to choose short video clips at a suitable level of difficulty to provide comprehensible input (Krashen, 1985), employ bilingual or pinyin-annotated subtitles to support listening comprehension, and create follow-up retrieval practice tasks based on the video material to enhance vocabulary retention.

Second, the direct effect of CMCP on BI is sufficiently strong to suggest that entertainment content has intrinsic motivational value for long-term learning engagement. Chinese-language learning materials created by YouTubers can also be enhanced with elements of popular Chinese entertainment to attract and retain Thai adolescent audiences.

Third, the robust BI → LE relationship implies that students’ developing intention to engage with Chinese media on YouTube is associated with greater self-perceived learning effectiveness. Educational stakeholders and curriculum designers can consider creating guidance materials to help students select appropriate Chinese-language content on YouTube and redirect their entertainment interests toward self-directed language learning.

### 4.6 Limitations

A number of limitations ought to be recognized. First, the study employed a cross-sectional design in which all variables were measured at a single point in time using self-report questionnaires. The relationships between the path variables in the structural model must thus be understood as predictive rather than causal. To determine the directionality of causation, longitudinal or experimental designs would be required.

Second, LE was assessed using self-reported perceptions of students and not objective language proficiency tests. It is important to note that self-perceived learning effectiveness reflects the perceived benefits (e.g., vocabulary, speaking, writing, listening) that are psychologically significant and can enhance learners’ perceived competence and language-learning self-efficacy (Bandura, 1997). Within the framework of informal learning, when standardized tests are generally absent, such perceived gains can serve as a proximal motivational outcome that sustains long-term engagement with target-language media (Pajares, 2003). Nevertheless, subjective benefits do not necessarily correspond to objective performance, and future research ought to triangulate self-reports with test-based or behavioral logs.

Third, the sample was selected based on purposive sampling in two secondary schools in Thailand, which may restrict the generalizability of the results to other areas, school types, or education levels. In future research, the samples should be more diverse to increase external validity.

Fourth, CMCP was measured using three items that assessed learners’ willingness to consume Chinese entertainment content. Although willingness is a behavioral disposition rather than an affective state in itself, it can serve as a behaviorally based proxy for the content-satisfaction dimension of UGT. Namely, the expressed willingness to use certain types of media indicates the underlying gratification-seeking orientation that UGT assumes as one of the sources of media choice (Katz et al., 1973). Nevertheless, further research may include more direct measures of affective engagement, such as enjoyment or flow, which would allow for a more comprehensive assessment of content-related gratification.

Fifth, PEOU was not included in the structural model. Although a single PEOU-related item was collected (Q22: ‘Using YouTube to learn Chinese is easy and convenient’; M = 3.86, SD = 1.12), the exclusion was a theoretical decision to maintain model parsimony and focus on the content-level pathway. The observed variance of this item suggests that PEOU may not have been range-restricted in this sample. Future research should incorporate PEOU to examine its potential role in the model.

Sixth, the CMCP construct was measured with three items, and the factor loading of Q13 (Chinese songs, 0.632) was notably lower than Q14 (0.810) and Q15 (0.908). Although all loadings exceeded the 0.50 threshold and the construct met standard convergent validity criteria (AVE = 0.627, CR = 0.831), the heterogeneity in loadings suggests that different media types may contribute unevenly to the overall content preference construct. A higher-order factor structure could not be tested due to the limited number of indicators. Future research should develop a more comprehensive, multi-dimensional scale for Chinese media content preference that captures a wider range of media types and incorporates direct measures of affective gratification, such as enjoyment and flow, to more fully reflect the core principles of UGT.

Seventh, the present study did not examine potential moderating effects of demographic variables such as age, grade level, HSK proficiency, or ethnic Chinese heritage. The feasibility of such analyses was limited by the uneven distribution of the sample: 90.6% of participants had not taken the HSK, Grade 10 had only one participant (reflecting the availability of Chinese-language classes during the data collection period), and age groups were unevenly distributed (43.23% aged 13–14 vs. 8.27% aged 18–19). These imbalances would result in insufficient cell sizes for meaningful multi-group SEM analyses. Future research with larger and more balanced samples should investigate whether the proposed pathways vary across learner subgroups.

### 4.7 Future research directions

In view of the above analysis, future research could extend this work in the following ways. First, moderating variables such as self-regulated learning ability, learning autonomy, or prior Chinese proficiency could be introduced to explore the model’s boundary conditions. In particular, variables collected but not modeled in this study, such as ethnic Chinese heritage and HSK proficiency level, could serve as moderators in future research with more balanced samples. This will clarify under which situations the predictive effects of PU on BI increase or decrease. Second, longitudinal or cross-lagged panel designs may be used to measure perceived usefulness, behavioral intention, and self-perceived learning effectiveness at different time points. This enhances causal inference and lessens the possible impact of shared method variance. Third, objective assessment measures such as HSK scores, classroom quiz scores, or platform usage logs can be incorporated to check the consistency between behavioral intention and actual learning behavior and results. Fourth, sample diversity could be broadened by including adult learners, students from other cultural backgrounds, or students from multiple schools and regions in Thailand.

## 5 Conclusion

This study has explored the processes by which the Chinese entertainment media preferences of Thai secondary school students are linked to their informal language learning on YouTube, using a combined TAM and UGT model. The results affirm that content-level gratification is an effective antecedent in the technology acceptance process: learners’ preference for Chinese songs, dramas, and variety shows encourages them to use YouTube as a Chinese learning platform, and this relationship is largely mediated by perceived usefulness rather than driven solely by direct hedonic motivation. The dominance of the cognitive evaluation pathway over the direct gratification pathway implies that adolescent learners do not simply consume entertainment passively but can convert affective engagement into rational judgments of instrumental value. In addition, behavioral intention was found to be a powerful predictor of self-perceived learning effectiveness, suggesting that sustained and purposeful engagement with target-language media is associated with stronger perceived improvement and greater confidence in Chinese learning. Taken together, the study expands TAM research into content-driven, non-formal learning settings and shows how TAM and UGT can be used together to explain such settings. The results offer practical recommendations to Chinese-language teachers who want to connect informal media consumption with formal teaching, to content developers who would like to create engaging, learning-focused content, and to policymakers who need to develop policies to support Chinese-language education in Thailand.

## Data Availability

The original contributions presented in the study are included in the article/Supplementary material, further inquiries can be directed to the corresponding author.

## Footnotes

1
www.wjx.cn
