# Supplementary material for: Integrating TAM and uses and gratifications theory: how Chinese media content preferences predict YouTube-based Chinese language learning among Thai secondary school students
Source: Front Psychol. 2026 May 19;17:1824902. doi: 10.3389/fpsyg.2026.1824902 (PMC13226547; doi:10.3389/fpsyg.2026.1824902)
Supplement: Supplementary file 1 [file Table_1.docx]

**Supplementary Material**

**Complete Survey Questionnaire (30 Items)**

**Response scales:**

Willingness items (CMCP, BI, Q7, Q9): 1 = Very unwilling, 2 = Unwilling, 3 = Neutral, 4 = Willing, 5 = Very willing

Agreement items (PU, LE, Q17–Q19, Q22, Q24–Q25, Q30): 1 = Strongly disagree, 2 = Disagree, 3 = Neutral, 4 = Agree, 5 = Strongly agree

Demographics (Q1–Q6): Categorical responses as indicated.

| **Item** | **Construct** | **Thai** | **Chinese** | **English** | **Used** |
| --- | --- | --- | --- | --- | --- |
| Q1 | Demographics | เพศของคุณคือ | 你的性别是？ | What is your gender? | No |
| Q2 | Demographics | อายุของคุณคือ | 你的年龄是? | What is your age? | No |
| Q3 | Demographics | ระดับการศึกษาของคุณ | 你读哪个年级? | What is your grade level? | No |
| Q4 | Demographics | ระยะเวลาที่คุณเรียนภาษาจีน | 你学汉语多久了? | How long have you studied Chinese? | No |
| Q5 | Demographics | ระดับภาษาจีนของคุณเป็นอย่างไร | 你的汉语水平怎么样? | What is your Chinese proficiency level? | No |
| Q6 | Demographics | คุณเป็นเชื้อสายจีนหรือไม่ | 你是否为华裔? | Are you of ethnic Chinese origin? | No |
| Q7 | Not used | คุณเต็มใจที่จะใช้โทรศัพท์หรืออุปกรณ์อิเล็กทรอนิกส์อื่นๆในการเรียนภาษาจีนหรือไม่ | 你愿意使用手机、电脑、平板等来学习中文吗? | Are you willing to use phones, computers, tablets, etc. to learn Chinese? | No |
| Q8 | BI | คุณเต็มใจใช้วีดีโอจากYouTubeในการเรียนภาษาจีนหรือไม่ | 你愿意使用YouTube视频来学习中文吗? | Are you willing to use YouTube videos to learn Chinese? | Yes |
| Q9 | Not used | คุณเต็มใจในการจ่ายค่าใช้จ่ายในการใช้วีดีโอจากYouTube | 你愿意付费观看YouTube视频学中文吗？ | Are you willing to pay to watch YouTube videos to learn Chinese? | No |
| Q10 | BI | คุณยินดีใช้วีดีโอจากYouTubeในการเรียนภาษาจีนต่อไปในอนาคตหรือไม่ | 你愿意未来继续看YouTube视频学中文吗？ | Are you willing to continue using YouTube to learn Chinese in the future? | Yes |
| Q11 | BI | คุณยินดีแนะนำให้คนอื่นใช้วีดีโอจากYouTubeในการเรียนภาษาจีนหรือไม่ | 你愿意推荐别人看YouTube视频学中文吗？ | Are you willing to recommend others to learn Chinese via YouTube? | Yes |
| Q12 | BI | คุณยินดีดูการเรียนการสอนภาษาจีนบนYouTubeหรือไม่ | 你愿意在YouTube上看专门的汉语教学视频吗？ | Are you willing to watch Chinese teaching videos on YouTube? | Yes |
| Q13 | CMCP | คุณยินดีฟังเพลงจีนบนYouTubeหรือไม่ | 你愿意在YouTube上听中文歌吗？ | Are you willing to listen to Chinese songs on YouTube? | Yes |
| Q14 | CMCP | คุณยังดีดูหนังละครภาษาจีนบนYouTubeหรือไม่ | 你愿意在YouTube上看中国电影和电视剧吗？ | Are you willing to watch Chinese movies/TV dramas on YouTube? | Yes |
| Q15 | CMCP | คุณยินดีดูรายการวาไรตี้ภาษาจีนบนYouTubeหรือไม่ | 你愿意在YouTube上看中国综艺节目吗？ | Are you willing to watch Chinese variety shows on YouTube? | Yes |
| Q16 | BI | คุณยินดีเรียนภาษาจีนผ่านYouTubeทุกวันหรือไม่ | 你愿意每天在YouTube上学中文吗？ | Are you willing to learn Chinese on YouTube every day? | Yes |
| Q17 | Not used | คุณชื่นชอบการเรียนภาษาจีน | 你喜欢学中文 | You like learning Chinese. | No |
| Q18 | Not used | คุณคิดว่าวีดีโอภาษาจีนบนYouTubeจำเป็นต้องมีเพิ่มมากขึ้น | 你认为现在YouTube上的中文视频还需更多 | You think there need to be more Chinese videos on YouTube. | No |
| Q19 | Not used | คุณชอบดูวีดีโอบนYouTube | 你喜欢用YouTube看视频 | You like watching videos on YouTube. | No |
| Q20 | PU | คุณคิดว่าการดูวีดีโอบนYouTubeสามารถทำให้ภาษาจีนของคุณก้าวหน้า | 你认为使用YouTube能让中文学习变得高效 | Using YouTube can make Chinese learning more efficient. | Yes |
| Q21 | PU | คุณคิดว่าการใช้YouTubeในการเรียนมีส่วนช่วยให้คะแนนภาษาจีนของคุณสูงขึ้น | 你认为使用YouTube学习能提高中文成绩 | Using YouTube for learning can improve my Chinese grades. | Yes |
| Q22 | Not used | คุณคิดว่าการเรียนภาษาจีนผ่านYouTubeง่ายและสะดวกสบาย | 你认为使用YouTube学中文操作简单、方便快捷 | Using YouTube to learn Chinese is easy and convenient. | No |
| Q23 | PU | คุณคิดว่าการใช้YouTubeเหมาะสมกับข้อกำหนดของการเรียนภาษาจีน | 你认为使用YouTube能满足中文学习需求 | Using YouTube can meet my Chinese learning needs. | Yes |
| Q24 | Not used | คุณชอบคุณครูที่สอนภาษาจีนบนYouTube | 你喜欢YouTube上教中文的老师 | You like the Chinese teachers on YouTube. | No |
| Q25 | Not used | คุณคิดว่าวีดีโอบนYouTubeมีความน่าสนใจอย่างมาก | 你认为YouTube上的中文视频很有趣 | You think the Chinese videos on YouTube are very interesting. | No |
| Q26 | LE | จากการดูวีดีโอภาษาจีนบนYouTube คุณรู้สึกว่าจำนวนคำศัพท์ที่คุณรู้มีมากขึ้น | 通过看YouTube的中文视频，你的词汇量增加了 | By watching YouTube Chinese videos, my vocabulary has increased. | Yes |
| Q27 | LE | จากการดูวีดีโอบนYouTube คุณรู้สึกว่าทักษะการพูดภาษาจีนของคุณมีความคล่องแคล่วยิ่งขึ้น | 通过看YouTube的中文视频，你的口语更流利了 | By watching YouTube Chinese videos, my speaking fluency has improved. | Yes |
| Q28 | LE | จากการดูวีดีโอบนYouTube คุณสามารถเขียนอักษรจีนได้มากขึ้น | 通过看YouTube的中文视频，你会写更多汉字了 | By watching YouTube Chinese videos, I can write more Chinese characters. | Yes |
| Q29 | LE | จากการดูวีดีโอบนYouTube คุณรู้สึกว่าทักษะการฟังภาษาจีนของคุณชัดเจนขึ้น | 通过看YouTube的中文视频，你听汉语更清晰了 | By watching YouTube Chinese videos, my listening comprehension has improved. | Yes |
| Q30 | Not used | คุณคิดว่าการใช้YouTubeมีส่วนช่วยในการเรียนภาษาจีนของคุณ | 你认为使用YouTube学习中文对你有帮助 | You think using YouTube to learn Chinese is helpful to you. | No |

***Note.*** “Used” column indicates whether the item was included in the structural equation model. Items marked “No” were administered but excluded from the final model. The questionnaire was administered in bilingual format (Chinese and Thai). BI = Behavioral Intention; CMCP = Chinese Media Content Preference; PU = Perceived Usefulness; LE = Self-Perceived Learning Effectiveness.
